# Patellofemoral Joint Outcomes in Kinematically vs. Mechanically Aligned Total Knee Arthroplasty: A Systematic Review and Meta-Analysis

**DOI:** 10.3390/medicina62071233

**Published:** 2026-06-26

**Authors:** Tanwir Gul, Prabhbir Singh Devgun, Sohaib Ahmed Feroz, Azka Syed, Sherif Isaac, Anastasios P. Nikolaides, Mohammed Elmajee

**Affiliations:** 1 University Hospitals Birmingham NHS Foundation Trust, Birmingham B15 2GW, UK; sohaibahmed.feroz@uhb.nhs.uk (S.A.F.);; 2 Stockport NHS Foundation Trust, Poplar Grove, Stockport SK2 7JE, UK; 3 Department of Biotechnology, University of Karachi, Karachi 75270, Pakistan; azkaassyed31@gmail.com; 4 Worcestershire Acute Hospitals NHS Trust, Charles Hastings Way, Worcester WR5 1DD, UK; sherif.isaac@nhs.net

**Keywords:** mechanical alignment, kinematic alignment, total knee arthroplasty, patellofemoral joint

## Abstract

*Background and Objectives*: While multiple studies have compared mechanical alignment (MA) and kinematic alignment (KA) in total knee arthroplasty (TKA), the effect of alignment philosophy on the patellofemoral joint (PFJ) remains unclear. This systematic review evaluates PFJ-related outcomes following KA- versus MA-TKA indirectly through functional knee scores. *Materials and Methods*: A comprehensive search of Ovid MEDLINE, PubMed Central, and Embase was undertaken in October 2025 using search strategies developed by two independent librarians. Screening and data extraction were performed independently by two reviewers. All primary studies, including randomised and non-randomised studies, comparing KA- and MA-TKA that included functional Knee Society Score (KSS-F) or the Knee Injury and Osteoarthritis Outcome Score (KOOS) Sports subscale were included. *Results*: Of the 1570 identified studies, 13 met the inclusion criteria for the systematic review and meta-analysis. KA demonstrated a significant improvement in KSS-F at 1-year follow-up compared with MA (*p* < 0.05). At longer follow-up intervals, differences did not reach statistical significance (*p* = 0.07); however, outcomes consistently favoured KA, suggesting maintenance of the early functional advantage over time. *Conclusions*: KA-TKA is associated with superior PFJ-related functional outcomes at 1 year and shows a sustained trend towards improved performance in longer-term follow-up based on the surrogate scores. Further prospective studies incorporating PFJ-sensitive outcome measures are warranted to clarify long-term benefits.

## 1. Introduction

Total knee arthroplasty (TKA) is the established treatment of choice for severe knee osteoarthritis. It is successful in relieving pain, restoring mobility, and improving the quality of life in a majority of patients. This has been shown through improvement in functional knee scores in countless studies [1]. Despite this, some patients continue to experience post-operative pain, instability, and dissatisfaction [2]. The causes are multifactorial, with knee alignment proposed as one of the factors that may be linked to post-operative outcomes.

Mechanical alignment (MA) is based on the principle that obtaining a neutral hip-knee-ankle (HKA) axis will result in symmetrical force distribution across the implant, thus reducing wear and improving survivorship [3]. Supported by long-term research, and being a standardised and reproducible procedure, it has remained the widely accepted method for TKA. However, the MA philosophy has been critiqued because up to 20% of patients remain dissatisfied post-operatively [2,4]. Most patients display a physiological degree of HKA varus or valgus [5]. MA philosophy fails to account for this and often requires extensive bone resections leading to alteration in native joint anatomy and knee kinematics. Furthermore, HKA is based on static radiographs and does not account for dynamic changes during gait. Ligament releases are often performed due to extensive bone cuts and may predispose patients to stiffness and instability [6]. In response to these limitations, kinematic alignment (KA) has emerged as an alternative philosophy. It is based on the principle that preserving the patient’s natural anatomy, accepting a degree of varus or valgus alignment, and minimising soft tissue release will maximise patient outcomes [7]. Although long-term data are limited, pooled data show comparable patient satisfaction without evidence of reduced implant survivorship [8,9].

Patellofemoral joint (PFJ) problems contribute to post-TKA dissatisfaction [10]. Knee alignment can impact PFJ function, which plays a pivotal role in load bearing, stair climbing, and knee extension. Changes in component orientation and soft tissue releases done in MA can disrupt patellar tracking and cause patellofemoral stress, contributing to post-operative anterior knee pain [11,12]. In contrast, several cadaveric studies suggest that KA may help to preserve natural PFJ biomechanics, although this remains an area of ongoing debate [13,14].

To date, no systematic review has specifically compared PFJ-related outcomes in MA- and KA-TKAs. The majority of studies report global knee scores, such as the Oxford Knee Score (OKS), Knee Society Score (KSS), and WOMAC [9]. These do not isolate PFJ-related outcomes, and thus PFJ dysfunction post-TKA may represent an under-recognised contributor to patient post-operative satisfaction. This systematic review aims to isolate PFJ outcome measures from composite scores and compares them between MA and KA techniques.

## 2. Materials and Methods

This systematic review was reported in line with the PRISMA (Preferred Reporting Items for Systematic Reviews and Meta-Analyses) guidelines [15] and prospectively registered with PROSPERO (registration number: CRD420251136625).

### 2.1. Search Strategy

The search strategy was conducted independently by two librarians from separate hospital trusts. The search results were then combined, and duplicate articles were removed before screening.

The first search strategy was formulated and kindly carried out by SC at University Hospitals Birmingham (UHB) trust on 10 October 2025. The second search strategy was performed by NM at Stockport NHS Foundation Trust on 23 October 2025. Although the search strategies addressed the same research question, the search term combinations differed between the two librarians. Full details of the search strategy can be found in the Supplementary Materials, Files S1 and S2.

Primary research databases including Ovid MEDLINE, PubMed Central and Embase were searched. A combination of free-text terms and Medical Subject Heading (MeSH) terms were used with Boolean operators. Searches were limited to human studies. See the Supplementary Materials (Files S1 and S2) for the full search strategy used.

### 2.2. Eligibility Criteria

#### 2.2.1. Inclusion Criteria

We included randomised controlled trials (RCTs) and cohort studies involving patients of all ages undergoing KA-TKA for osteoarthritis (OA) of the knee. Only primary studies that compared the outcomes of KA-TKA to MA-TKA were included in this systematic review. The outcomes of interest were Sport and Recreation Function subsection of the Knee Injury and Osteoarthritis Outcome Score (KOOS) and the Function subsection of the Knee Society Score (KSS-F).

There was no restriction placed on the publication date, implant type, minimum follow-up duration or whether the disease was unilateral or bilateral. Only articles in the English language were included in this systematic review. However, the language restriction was not implemented in the title/abstract screening stage to check for availability of a translated version of the article. This was to ensure articles were not excluded prematurely.

#### 2.2.2. Exclusion Criteria

Any cadaveric studies, computational model studies, systematic reviews, meta-analyses, editorials, expert opinions, conference abstracts, and case reports were excluded. For studies that included the KOOS or the KSS scores but did not include a breakdown with the specific subsections of interest, the authors were emailed to request these data. If the relevant data could not be obtained, the study was excluded.

### 2.3. Study Selection and Data Extraction

The Rayyan AI (Rayyan Systems, Inc., Cambridge, MA, USA) website was used for the screening process (https://www.rayyan.ai, accessed on 21 September 2025). Title and abstract screening and then full text screening were performed by two independent reviewers (TG and PD). Any discrepancies in the decisions were discussed and resolved amongst the two independent authors. If the two authors could not come to an agreement, then the senior author (ME) was consulted to come to a consensus.

A data extraction table was created by ME in Google Sheets (Google LLC, Mountain View, CA, USA) prior to the data collection stage. A separate data extraction form was created by the statistician (AS), which focused more on the quantitative data. The data from the selected studies were extracted with help from multiple reviewers (TG, SF, PD, AS). In addition to primary outcome measures, information on study design, sample size, age, sex and follow-up duration was also collected (see Table 1).

Mean and standard deviation (SD) were extracted directly from each included study. When outcomes were reported graphically, SDs were estimated using Graphreader (Graphreader V2, Version 1.0) (https://www.graphreader.com/, accessed on 15 January 2026). For studies reporting median and interquartile range (IQR), values were converted to mean and SD using the method described by Wan et al. [16].

### 2.4. Outcomes of Interest

#### 2.4.1. Knee Injury and Osteoarthritis Outcome Score (KOOS)

The Knee Injury and Osteoarthritis Outcome Score (KOOS) is a questionnaire developed in the 1990s to assess short-term and long-term outcomes in patients following knee injury [17]. It has since been validated in different population groups, including subjects undergoing TKA for OA [18]. The KOOS questionnaire measures outcomes on five different subscales: Pain, Symptoms, ADL Function, Sport and Recreation Function, and Quality of Life. Of the five subsections of the KOOS questionnaire, Sport and Recreation Function has the greatest number of items related to the PFJ outcomes. Hence, this study focused on the Sports and Recreation Function subsection of the KOOS.

#### 2.4.2. Knee Society Score (KSS)

The KSS is a tool developed in 1989 for measuring objective and subjective outcomes pre- and post-operatively in patients undergoing TKA [19]. The KSS was revised in 2011, resulting in the current version [20,21]. The KSS includes subsections on Symptoms, Patient Satisfaction, Patient Expectation, and Functional Activities. From these subsections, Functional Activities has the highest proportion of items on PFJ-related outcomes. Hence, this study focuses on the functional subsection of the KSS scoring system.

### 2.5. Study Appraisal

All included studies were critically appraised using the Critical Appraisal Skills Programme (CASP) checklist tool [22].

### 2.6. Meta-Analysis

A meta-analysis was undertaken to compare post-operative KSS-F in KA-TKA and MA-TKA. Continuous outcomes were synthesised as standardised mean differences (SMDs) with corresponding 95% confidence intervals (CIs) using the inverse-variance approach. In anticipation of clinical and methodological heterogeneity across the included studies, a random-effects model was applied when substantial heterogeneity was observed (I^2^ > 50%), whereas a fixed-effect model was used when heterogeneity was low (I^2^ ≤ 50%). All statistical analyses were performed using Review Manager (RevMan) software, version 5.4 (The Cochrane Collaboration, London, UK). Statistical heterogeneity was evaluated using Cochran’s Q (χ^2^) test, the I^2^ statistic, and between-study variance (τ^2^). Pre-specified subgroup analyses were conducted according to duration of follow-up (6 months, 1 year, and 2 years). Statistical significance was defined as a two-sided *p* value of less than 0.05. Additional sensitivity analyses were performed by restricting the analysis to randomised controlled trials and studies published between 2023 and 2025.

## 3. Results

### 3.1. Study Selection

**Figure 1:**
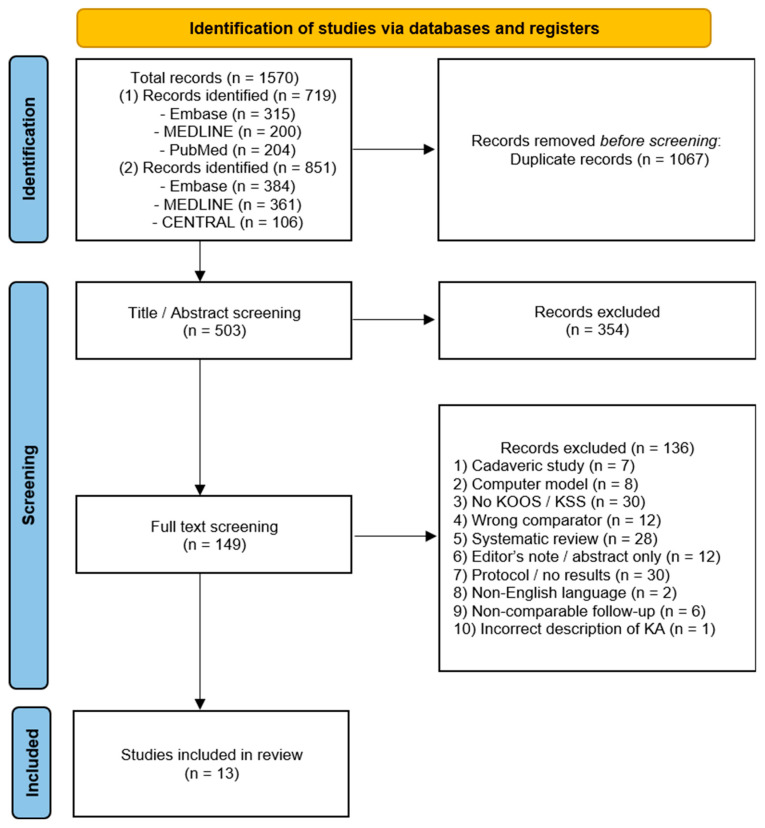
PRISMA flow chart.

A total of 1570 articles were identified from our initial search. After de-duplication, the remaining 503 had their titles and abstracts screened. A total of 354 records were excluded, leaving 149 articles. Ultimately, 13 articles were included after full text screening (Figure 1). For studies that included the KOOS or KSS score without the relevant subsection of interest, the corresponding authors were contacted to obtain the data of interest. A total of 11 authors were emailed, of which one replied with the data of interest, resulting in that study being included. Another author replied confirming not having the data, and hence the study was excluded along with those whose authors did not respond.

### 3.2. Study Characteristics

A summary of the demographics and characteristics of the included study can be found in Table 1 below. The table outlines the study design, sample size, age, sex, follow-up duration and outcome measures of all the included studies.

**Table 1:** Summary of study characteristics. RCT—randomised controlled trial; MA—mechanical alignment; KA—kinematic alignment; KSS-F—functional subscale of knee society score; KOOS—knee injury and osteoarthritis outcome score.

Study	Study Design	Sample Size (MA/KA)	Mean Age in Years (MA/KA)	Male Sex (MA/KA)	Follow-Up Duration (Years)	Outcome Measure
Koutp et al., 2025 [23]	RCT	5/50	69.60 ± 9.30/72.52 ± 8.57	20/21	1, 2	KSS-F
Franceschetti et al., 2025 [24]	Retrospective cohort study	200/132	70.3 ± 7.1/70.1 ± 7.0	85/57	1	KSS-F
Bauer et al., 2025 [25]	RCT	4/41	Not reported	Not reported	1	KSS-F
Lychagin et al., 2025 [26]	RCT	49/47	67 ± 9.0/66 ± 7.0	13/12	0.5, 1	KSS-F
Ettinger et al., 2024 [27]	RCT	51/47	63.1 ± 13.4/68.8 ± 9.9	31/29	1, 2	KSS-F
Wen et al., 2023 [28]	Retrospective cohort study	61/65	71.2 ± 7.1/70.6 ± 6.4	21/19	2	KSS-F
Kim et al., 2022 [29]	Retrospective cohort study	126/42	70.38 ± 5.76/70.30 ± 5.06	9/2	2	KSS-F
Koh et al., 2021 [30]	Prospective cohort study	93/93	67.0 ± 7.4/69.1 ± 8.1	20/23	0.5, 2	KSS-F
Abhari et al., 2021 [31]	Retrospective cohort study	115/109	66 ± 0.93/67 ± 0.90	44/63	1	KSS-F
Jeremić et al., 2020 [32]	Prospective case-control study	24/24	72.5 ± 5.8/70.7 ± 6.7	11/11	1	KSS-F + KOOS Sports
Young et al., 2017 [33]	RCT	50/49	70 ± 7.5/72 ± 6.5	24/24	2	KSS-F
Dossett et al., 2014 [34]	RCT	60/60	66 ± 8.6/66 ± 7.7	38/41	1	KSS-F
Rosa et al., 2025 [35]	RCT	50/59	66 ± 7.42/66 ± 7.74	26/24	2	KOOS Sports

### 3.3. KSS Function

**Figure 2:**
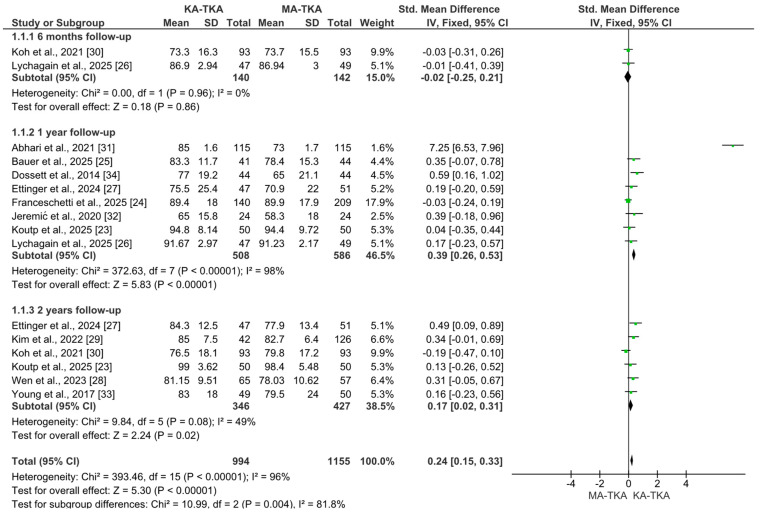
Forest plot showing the quantitative synthesis of KSS-F scores comparing MA-TKA and KA-TKA at 6 months, 1 year, and 2 years’ follow-up [23,24,25,26,27,28,29,30,31,32,33,34].

Twelve studies were included in the quantitative synthesis of KSS-F, comprising two reports at 6 months, eight reports at 1 year, and six reports at 2 years’ follow-up; several studies contributed data to more than one follow-up time point (Koutp et al., 2025 [23]; Franceschetti et al., 2025 [24]; Bauer et al., 2025 [25]; Lychagin et al., 2025 [26]; Ettinger et al., 2024 [27]; Wen et al., 2023 [28]; Kim et al., 2022 [29]; Koh et al., 2021 [30]; Abhari et al., 2021 [31]; Jeremić et al., 2020 [32]; Young et al., 2017 [33]; Dossett et al., 2014 [34]). Pooled analysis using a random-effects model of 1155 knees in the MA-TKA group and 994 knees in the KA-TKA group demonstrated statistically significant improvement in KSS-F favouring KA over MA (SMD = 0.59, 95% CI 0.12 to 1.07; Z = 2.47; *p* = 0.01). However, considerable between-study heterogeneity was present (I^2^ = 96%), indicating substantial variability in reported functional outcomes across studies (Figure 2).

#### 3.3.1. Six-Month Follow-Up

Two studies, including 282 knees, reported KSS-F at six months (Lychagin et al., 2025 [26]; Koh et al., 2021 [30]). The pooled estimate demonstrated no difference between KA and MA at this early time point (SMD = −0.02, 95% CI −0.25 to 0.21; Z = 0.18; *p* = 0.86). No heterogeneity was detected (I^2^ = 0%), indicating highly consistent short-term results.

#### 3.3.2. One-Year Follow-Up

Eight studies comprising 1094 knees contributed data at one year (Franceschetti et al., 2025 [24]; Koutp et al., 2025 [23]; Lychagin et al., 2025 [26]; Bauer et al., 2025 [25]; Ettinger et al., 2024 [27]; Abhari et al., 2021 [31]; Jeremić et al., 2020 [32]; Dossett et al., 2014 [34]). The pooled analysis demonstrated a statistically significant improvement in KSS-F favouring KA (SMD = 1.09, 95% CI 0.07 to 2.11; Z = 2.09; *p* = 0.04). However, marked heterogeneity was evident (I^2^ = 98%), suggesting substantial variability in effect estimates across studies at this follow-up interval.

#### 3.3.3. Two-Year Follow-Up

Six studies involving 751 knees were included in the two-year subgroup (Koutp et al., 2025 [23]; Ettinger et al., 2024 [27]; Wen et al., 2023 [28]; Kim et al., 2022 [29]; Koh et al., 2021 [30]; Young et al., 2017 [33]). Although the pooled mean difference numerically favoured KA, this did not reach statistical significance (SMD = 0.19, 95% CI −0.02 to 0.40; Z = 1.80; *p* = 0.07). Moderate heterogeneity was observed (I^2^ = 49%), indicating some between-study variability, but with greater consistency than observed at one year.

#### 3.3.4. Sensitivity Analysis

**Figure 3:**
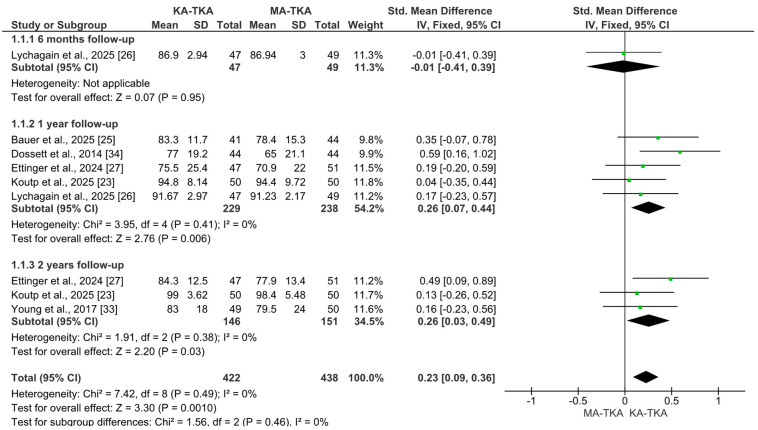
Sensitivity analysis including randomised controlled trials (RCTs) only—forest plot of standardised mean differences (SMD) in KSS-F comparing KA-TKA with MA-TKA [23,25,26,27,33,34].

When analysis is restricted to high-quality RCT evidence comprising 860 knees (KA: 422; MA: 438), the pooled analysis demonstrated a consistent trend favouring KA-TKA over MA-TKA across follow-up intervals (Figure 3).

At 6 months, there was no significant difference between groups (SMD −0.01, 95% CI −0.41 to 0.39; *p* = 0.95), based on two reports including 47 KA-TKA and 49 MA-TKA knees.

At 1 year, a statistically significant improvement in functional outcomes was observed in the KA-TKA cohort (SMD 0.26, 95% CI 0.07 to 0.44; *p* = 0.006). This analysis included 229 KA-TKA and 238 MA-TKA knees. Heterogeneity was negligible (I^2^ = 0%), indicating consistency across studies.

Similarly, at 2 years’ follow-up, KA-TKA maintained a significant functional advantage (SMD 0.26, 95% CI 0.03 to 0.49; *p* = 0.03), derived from 146 KA-TKA and 151 MA-TKA knees, again with no observed heterogeneity (I^2^ = 0%).

Overall, pooling all time points (422 KA-TKA vs. 438 MA-TKA knees) demonstrated a modest but statistically significant improvement in KSS-F favouring KA (SMD 0.23, 95% CI 0.09 to 0.36; *p* = 0.001), with no meaningful heterogeneity (I^2^ = 0%). There was no significant subgroup difference between follow-up intervals (*p* = 0.46), suggesting the effect of KA was consistent across time points.

**Figure 4:**
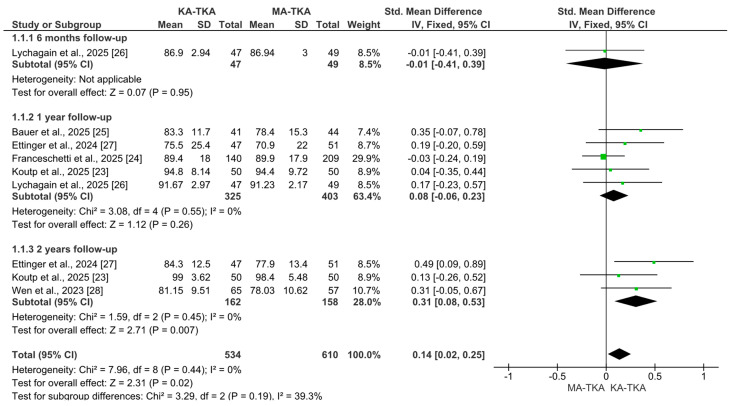
Forest plot of standardised mean differences (SMD) in KSS-F reported in recently published studies (2023–2025) comparing KA-TKA with MA-TKA [23,24,25,26,27,28].

When analysis is restricted to the literature published between 2023 and 2025, the forest plot demonstrated a consistent trend favouring KA-TKA (Figure 4). Although the magnitude and statistical significance varied across follow-up intervals. Early (<2-year) results suggested equivalence or a small, non-significant trend favouring KA (*p* < 0.05). At two years, KA-TKA conferred a functional advantage over MA-TKA (*p* = 0.007). The overall pooled analysis across all time points demonstrated a statistically significant (*p* = 0.02), albeit modest, improvement in KSS-F with KA-TKA. Heterogeneity appeared negligible, indicating that the findings were consistent across included studies and not driven by outliers.

### 3.4. KOOS Sports

**Figure 5:**
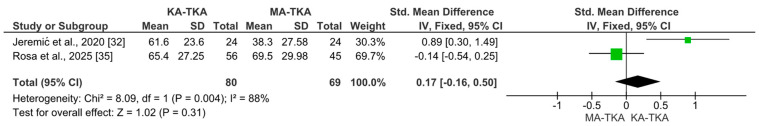
Forest plot showing the quantitative synthesis of KOOS Sports scores comparing MA-TKA and KA-TKA [32,35].

Two studies, including 149 knees, reported KOOS Sports at 1 year (Jeremić et al., 2020 [32]) and 2-years’ follow-up (Rosa et al., 2025 [35]). The pooled estimate demonstrated no difference between KA and MA across the included follow-up periods (SMD = 0.17 95% CI -0.16 to 0.50; Z = 1.02; *p* = 0.31). High heterogeneity was detected (Chi^2^ = 8.09, df = 1, *p* = 0.004; I^2^ = 88%), indicating highly inconsistent results (Figure 5).

## 4. Discussion

The PFJ has historically received less attention than tibiofemoral outcomes when evaluating functional recovery following recovery from TKA. While some recent studies have reported outcomes of KA- versus MA-TKA, they primarily evaluate global knee score rather than PFJ-related outcomes [9]. This systematic review and meta-analysis evaluated the effect of alignment philosophy on post-operative PFJ function, approximated using the KSS-F, which includes activities that impose significant PFJ loading such as stair climbing and squatting. The pooled analysis favoured KA over MA in terms of improvement in KSS-F scores (SMD 0.46; 95% CI 0.08–0.87; *p* = 0.02), although substantial heterogeneity was observed between studies (I^2^ = 95%). This suggests there was considerable variability in study populations, surgical techniques, and outcome assessment methods. Subgroup analysis demonstrated a statistically significant advantage for KA at 1 year (*p* < 0.05), whereas at 2 years the pooled estimate favoured KA but did not reach statistical significance.

Our findings align with evidence that KA-TKA achieves comparable functional outcomes to medial unicompartmental knee arthroplasty while more closely restoring physiological knee kinematics. These approaches share a focus on preserving native joint line orientation and soft tissue balance, which are critical for PFJ biomechanics. This supports the principle that the restoration of patient-specific anatomy, rather than adherence to a neutral mechanical axis, is central to optimising functional recovery and reducing PFJ symptoms following TKA [36].

The improved KSS-F outcomes observed at 1 year in the KA cohort may be related to more favourable PFJ biomechanics resulting from restoration of the patient’s native knee anatomy. KA has been shown to reduce the quadriceps force required for knee flexion in comparison with MA [37]. Given that quadriceps weakness is common in patients with OA, a reduction in quadriceps workload may facilitate earlier functional recovery and potentially reduce anterior knee pain following TKA [37]. Furthermore, KA more closely restores native trochlear morphology by maintaining the patient’s native femoral joint line orientation, which may help preserve physiological Q-angle mechanics and patellar tracking [38,39]. It has been noted, however, that most currently available implants are designed according to MA principles, suggesting that implant designs specifically optimised for KA principles may further improve PFJ outcomes [40].

Initially, the KA philosophy has faced criticism, particularly in cases involving restoration of extreme constitutional varus or valgus alignment. Concerns were raised that reproducing such alignment patterns may increase implant wear or long-term mechanical failure [41]. However, studies assessing intraoperative compartmental forces have not demonstrated a significant difference between KA and MA knees [42]. Another concern relates to implant design. One study reported that KA may result in increased lateral patellar tilt, which has been theorised to occur due to the use of femoral components originally designed for MA-TKA [30]. While implants specifically tailored for KA principles may further optimise PFJ mechanics, this remains an area that requires further investigation.

In contrast to the improvements observed at later follow-up intervals, early post-operative outcomes appear less sensitive to alignment strategy. One study included in this review demonstrated no significant difference in PFJ outcomes between MA- and KA-TKA at 6 months post-operatively. This likely reflects the predominance of early post-operative recovery processes. During the early post-operative period, functional outcomes are strongly influenced by factors such as soft tissue healing, inflammation, quadriceps inhibition, and rehabilitation progress [43,44,45]. In this phase, neuromuscular impairment and pain may limit functional performance to a degree that obscures more subtle biomechanical advantages associated with alignment technique. In addition, a potential ceiling effect of early outcome measures may further reduce the ability to detect differences between groups. It is therefore plausible that the biomechanical benefits of KA require sufficient neuromuscular recovery and strength restoration before translating into measurable improvements in PFJ-related function.

It is also important to note that only two studies contributed data for the 6-month follow-up analysis in this meta-analysis. Although the findings of these studies were concordant, the small number of contributing studies limits the robustness of this observation. This restricts the ability to generalise conclusions regarding early post-operative PFJ outcomes following TKA.

Several limitations should be acknowledged when interpreting the results of this study. Firstly, there is no universally accepted method for evaluating PFJ function following TKA [46]. As a result, composite knee scores such as the KSS and the KOOS are frequently used to indirectly assess PFJ-related outcomes [17,21]. In this systematic review, KSS-F was utilised as it captures activities that place significant load across the PFJ, including stair climbing and squatting. However, this approach has inherent limitations, as these measures are not specifically designed to evaluate PFJ symptoms. Several outcome measures have been developed specifically to assess PFJ-related symptoms, including the KOOS patellofemoral subscale (KOOS-PF) and the Kujala Anterior Knee Pain Score (AKPS) [47,48]. These instruments may provide greater sensitivity for detecting PFJ-related symptoms following TKA. However, their use in primary studies comparing KA and MA techniques remains limited. Restricting the analysis to studies that utilised PFJ-specific outcome measures would have substantially reduced the available evidence base, particularly given that KA is a relatively new surgical technique. Consequently, the use of surrogate measures of PFJ function represents a methodological limitation of this review. Future primary studies should incorporate PFJ-specific outcome measures to more accurately evaluate PFJ outcomes between different alignment strategies.

An additional limitation of this systematic review is the high heterogeneity observed at the one-year follow-up and in the overall analysis. This is likely attributed to the inclusion of non-randomised studies, as heterogeneity was negligible in the sensitivity analysis restricted to randomised controlled trials. Although the results of sensitivity analysis continued to favour KA-TKA, the observed effect size was smaller, suggesting that the overall findings should be interpreted with caution as the clinical significance remains uncertain. Another factor contributing to heterogeneity in the present meta-analysis is the variation in the implementation of KA techniques. No restrictions were placed on the type of KA technique used; therefore, both restricted and unrestricted KA studies were included [49,50]. Furthermore, no restrictions were applied regarding the method used to perform KA, meaning that studies utilising robotic-assisted [51], computer navigation-assisted [52], and calliper-verified [53] techniques were all included in the analysis. Although these techniques share the overarching philosophy of restoring patient-specific constitutional alignment [50], differences in surgical execution and accuracy may influence the reproducibility of patient outcomes. Finally, no objective measures of knee analysis were used, such as gait assessment or radiological assessment for patellar tracking. These factors likely contributed to statistical and clinical heterogeneity, and therefore, the findings should be interpreted with caution. Additionally, relatively short follow-up duration limits the ability to interpret the long-term impact of alignment philosophy on PFJ-related outcomes. Ideally, comparative analyses evaluating a single, standardised KA technique with long follow-up duration would reduce clinical heterogeneity and improve the internal validity of pooled analyses. However, imposing such restrictions would have substantially reduced the available evidence base, particularly given that KA remains an emerging surgical approach that has not yet been universally adopted. Limiting the inclusion criteria in this manner would therefore have compromised the feasibility of the meta-analysis. Consequently, all variants of KA were included in this review. The observed heterogeneity may therefore be partly explained by variation in KA techniques, which may introduce additional confounding factors and contribute to variability in patient-reported outcomes. These factors should be considered when interpreting the findings of the present study.

From a clinical perspective, this systematic review suggests that alignment philosophy may have an impact on PFJ-related outcomes. While the observed effects may be modest and interpreted with caution due to heterogeneity, the findings support the theory that restoring pre-arthritic knee kinematics may have functional relevance. This review also highlights that future research should aim to evaluate patellofemoral outcomes using PFJ-specific scoring systems and incorporate radiographic assessment methods that directly evaluate patellar tracking and alignment under physiological loading conditions. Imaging modalities such as the weight-bearing Merchant view may provide additional insight into PFJ biomechanics following TKA and help clarify the impact of alignment philosophy on PFJ function [54].

## 5. Conclusions

This systematic review and meta-analysis suggests that KA may be associated with improved PFJ functional outcomes compared with MA following TKA, based on indirect assessment using composite functional outcome measures. The pooled analysis identified a statistically significant improvement favouring KA, with subgroup analysis demonstrating a significant benefit at 1 year and a favourable trend persisting at 2 years. These findings support the concept that restoration of patient-specific knee anatomy and kinematics may improve PFJ biomechanics and functional recovery after arthroplasty.

While heterogeneity between studies and the limited use of PFJ-specific outcome measures represent important limitations, the consistency of the observed effect across studies suggests that alignment philosophy may meaningfully influence PFJ function following TKA. However, these findings should be interpreted with caution. Further high-quality studies incorporating PFJ-specific outcome measures, radiological assessment of patellar tracking, and longer-term follow-up are required to confirm these findings.

## Data Availability

The data used in the study for analysis are available from the corresponding author on reasonable request.

## Supplementary Materials

The following supporting information can be downloaded at: https://www.mdpi.com/article/10.3390/medicina62071233/s1, File S1: UHB Full Search Strategy; File S2: Stockport Full Search Strategy.

## Abbreviations

The following abbreviations are used in this manuscript:

MA	mechanical alignment
KA	kinematic alignment
TKA	total knee arthroplasty
PFJ	patellofemoral joint
KSS	knee society score
KOOS	knee injury and osteoarthritis outcome score
