# Supplementary material for: Patellofemoral Joint Outcomes in Kinematically vs. Mechanically Aligned Total Knee Arthroplasty: A Systematic Review and Meta-Analysis
Source: Medicina (Kaunas). 2026 Jun 26;62(7):1233. doi: 10.3390/medicina62071233 (PMC13413443; doi:10.3390/medicina62071233)
Supplement: Supplementary file 1 [file medicina-62-01233-s001.zip › Supplementary Materials - File S1.pdf]

Supplementary Material – S1: **UHB Full Search Strategy**

Kinematic alignment Vs Mechanical alignment in TKR- Systematic Review

Evidence search report

Completed: 10th October, 2025

If you would like to discuss the findings below or require an additional search, please contact: University Hospitals Birmingham (UHB) Library and Knowledge Service at semanti.chakraborty@uhb.nhs.uk

Please acknowledge this work in any resulting paper or presentation as:
Evidence search: Kinematic alignment Vs Mechanical alignment in TKR- Systematic Review. Semanti Chakraborty. 10th October, 2025. BIRMINGHAM, UK: University Hospitals Birmingham (UHB) Library and Knowledge Service.

A. Search terms and notes

Keywords, MESH terms used:

"Kinematic alignment", "Kinematically aligned", "Unrestricted Kinematic alignment", "Restricted Kinematic alignment", KA, "mechanical alignment", "mechanically aligned", MA, Arthroplasty, Replacement, Knee/ , total knee arthroplasty/, knee replacement/ , knee arthroplasty/, "total knee replacement", "knee replacement", "Knee Arthroplasty", TKA, TKR

For PRISMA 2020 flowchart:

Records Identified from:

Embase: 315

Medline: 200

Pubmed: 204

Total records before deduplication: 719

Records after duplicates removed:

Embase 257

Ovid MEDLINE 74

PubMed 31

Records Screened: 362

Population: Patients undergoing primary total knee replacement/arthroplasty (TKR/TKA)

Intervention: Kinematic Alignment (KA) Technique

Comparison: Mechanical Alignment (MA) Technique

Outcomes: Patient-Reported Outcome Measures (PROMs), Functional Outcomes, Patient Satisfaction, Clinical Outcomes, Radiological Outcomes, Surgical Outcomes, Complications and Safety

Limits: Human

For full search strategy see Section D below.

Please acknowledge this work in any resulting paper or presentation as:
Evidence search: Kinematic alignment Vs Mechanical alignment in TKR- Systematic Review. Semanti Chakraborty. 10th October, 2025. BIRMINGHAM, UK: University Hospitals Birmingham (UHB) Library and Knowledge Service.

B. How to access full content

Links are given to full text resources where available. For some of the papers, you will need an **NHS OpenAthens Account**. If you do not have an account you can register online.

You can then access the papers by simply entering your username and password. If you do not have easy access to the internet to gain access, please let us know and we can download the papers for you.

D. Search strategy

Ovid MEDLINE(R) ALL <1946 to October 08, 2025>

1 "Kinematic alignment".ab. or "Kinematic alignment".ti. 417
2 "Kinematically aligned".ab. or "Kinematically aligned".ti. 210
3 KA.ab. or KA.ti. 30161
4 "Unrestricted Kinematic alignment".ab. or "Unrestricted Kinematic alignment".ti. 25
5 "Restricted Kinematic alignment".ab. or "Restricted Kinematic alignment".ti. 61
6 ((Kinematic adj3 alignment).ab. or Kinematic.mp.) adj3 alignment.ti. [mp=title, book title, abstract,

original title, name of substance word, subject heading word, floating sub-heading word, keyword heading word, organism supplementary concept word, protocol supplementary concept word, rare disease supplementary concept word, unique identifier, synonyms, population supplementary concept word, anatomy supplementary concept word] 286
7 ((Kinematic adj3 aligned).ab. or Kinematic.mp.) adj3 aligned.ti. [mp=title, book title, abstract, original title, name of substance word, subject heading word, floating sub-heading word, keyword heading word, organism supplementary concept word, protocol supplementary concept word, rare disease supplementary concept word, unique identifier, synonyms, population supplementary concept word, anatomy supplementary concept word] 36
8 1 or 2 or 3 or 4 or 5 or 6 or 7 30471
9 "mechanical alignment".ab. or "mechanical alignment".ti. 843
10 MA.ab. or MA.ti. 99835
11 "mechanically aligned".ab. or "mechanically aligned".ti. 225
12 ((mechanically adj3 aligned).ab. or mechanically.mp.) adj3 aligned.ti. [mp=title, book title, abstract, original title, name of substance word, subject heading word, floating sub-heading word, keyword heading word, organism supplementary concept word, protocol supplementary concept word, rare disease supplementary concept word, unique identifier, synonyms, population supplementary concept word, anatomy supplementary concept word] 83
13 ((mechanically adj3 alignment).ab. or mechanically.mp.) adj3 alignment.ti. [mp=title, book title, abstract, original title, name of substance word, subject heading word, floating sub-heading word, keyword heading word, organism supplementary concept word, protocol supplementary concept word, rare disease supplementary concept word, unique identifier, synonyms, population supplementary concept word, anatomy supplementary concept word] 14
14 9 or 10 or 11 or 12 or 13 100629
15 exp Arthroplasty, Replacement, Knee/ 36497
16 "total knee replacement".ab. or "total knee replacement".ti. 6667
17 "knee replacement".ab. or "knee replacement".ti. 10704
18 "Knee Arthroplasty".ab. or "Knee Arthroplasty".ti. 37430
19 TKA.ab. or TKA.ti. 20332
20 TKR.ab. or TKR.ti. 2778
21 15 or 16 or 17 or 18 or 19 or 20 53193
22 8 and 14 and 21 298
23 limit 22 to humans 200

Embase <1974 to 2025 October 07>

1 "Kinematic alignment".ab. or "Kinematic alignment".ti. 443
2 KA.ab. or KA.ti. 20182
3 "Unrestricted Kinematic alignment".ab. or "Unrestricted Kinematic alignment".ti. 27
4 "Restricted Kinematic alignment".ab. or "Restricted Kinematic alignment".ti. 60
5 "Kinematically aligned".ab. or "Kinematically aligned".ti. 222
6 ((Kinematic adj3 alignment).ab. or Kinematic.mp.) adj3 alignment.ti. [mp=title, abstract, heading word, drug trade name, original title, device manufacturer, drug manufacturer, device trade name, keyword heading word, floating subheading word, candidate term word] 314
7 ((Kinematic adj3 aligned).ab. or Kinematic.mp.) adj3 aligned.ti. [mp=title, abstract, heading word, drug trade name, original title, device manufacturer, drug manufacturer, device trade name, keyword heading word, floating subheading word, candidate term word] 41
8 1 or 2 or 3 or 4 or 5 or 6 or 7 20527
9 "mechanical alignment".ab. or "mechanical alignment".ti. 939
10 MA.ab. or MA.ti. 121848
11 "mechanically aligned".ab. or "mechanically aligned".ti. 255
12 ((mechanically adj3 aligned).ab. or mechanically.mp.) adj3 aligned.ti. [mp=title, abstract, heading word, drug trade name, original title, device manufacturer, drug manufacturer, device trade name, keyword heading word, floating subheading word, candidate term word] 91
13 ((mechanically adj3 alignment).ab. or mechanically.mp.) adj3 alignment.ti. [mp=title, abstract, heading word, drug trade name, original title, device manufacturer, drug manufacturer, device trade name,

keyword heading word, floating subheading word, candidate term word] 13
14 9 or 10 or 11 or 12 or 13 122728
15 exp knee replacement/ 53866
16 exp total knee arthroplasty/ 45311
17 exp knee arthroplasty/ 70791
18 TKA.ab. or TKA.ti. 23759
19 TKR.ab. or TKR.ti. 4605
20 15 or 16 or 17 or 18 or 19 72700
21 8 and 14 and 20 322
22 limit 21 to human 315

PUBMED:

("Kinematic alignment"[All Fields] OR "Kinematically aligned"[All Fields]) AND ("mechanical alignment"[All Fields] OR "mechanically aligned"[All Fields]) AND ("total knee replacement"[All Fields] OR ("arthroplasty, replacement, knee"[MeSH Terms] OR ("arthroplasty"[All Fields] AND "replacement"[All Fields] AND "knee"[All Fields]) OR "knee replacement arthroplasty"[All Fields] OR ("total"[All Fields] AND "knee"[All Fields] AND "arthroplasty"[All Fields]) OR "total knee arthroplasty"[All Fields]) OR "TKA"[All Fields] OR "TKR"[All Fields]) Limits Applied: Human 204

E. Disclaimer

We hope that you find the evidence search service useful. Whilst care has been taken in the selection of the materials included in this evidence search, the Library and Knowledge Service is not responsible for the content or the accuracy of the enclosed research information. Accordingly, whilst every endeavour has been undertaken to execute a comprehensive search of the literature, the Library and Knowledge Service is not and will not be held responsible or liable for any omissions to pertinent research information not included as part of the results of the enclosed evidence search. Users are welcome to discuss the evidence search findings with the librarian responsible for executing the search. We welcome suggestions on additional search strategies / use of other information resources for further exploration. You must not use the results of this search for commercial purposes. Any usage or reproduction of the search output should acknowledge the Library and Knowledge Service that produced it. Search outputs may be published on KnowledgeShare for circulation to other healthcare staff and students. The original requester's name is not associated with the output unless permission is given.

Please acknowledge this work in any resulting paper or presentation as:
Evidence search: Kinematic alignment Vs Mechanical alignment in TKR- Systematic Review. Semanti Chakraborty. 10th October, 2025. BIRMINGHAM, UK: University Hospitals Birmingham (UHB) Library and Knowledge Service.
