# Supplementary material for: Patellofemoral Joint Outcomes in Kinematically vs. Mechanically Aligned Total Knee Arthroplasty: A Systematic Review and Meta-Analysis
Source: Medicina (Kaunas). 2026 Jun 26;62(7):1233. doi: 10.3390/medicina62071233 (PMC13413443; doi:10.3390/medicina62071233)
Supplement: Supplementary file 1 [file medicina-62-01233-s001.zip › Supplementary Materials - File S2.pdf]

Supplementary Material – S2: **Stockport Full Search Strategy**

Kinematic vs mechanical alignment in total knee replacement

Evidence search report

Completed: 23rd October, 2025

If you would like to discuss the findings below or require an additional search, please contact: Stockport NHS Knowledge and Library Services at Library.Enquiries@stockport.nhs.uk

Please acknowledge this work in any resulting paper or presentation as:
Evidence search: Kinematic vs mechanical alignment in total knee replacement. Naomi Majek. 23rd October, 2025. UK: Stockport NHS Knowledge and Library Services.

Summary of results

I have attached the results separately as RIS files. Total number of results from each database:

MEDLINE - 361

Embase - 384

CENTRAL (Cochrane Central Register of Controlled Trials) - 106

Contents

A. Search terms and notes

B. How to access full text

C. Search results

D. Search strategy

E. Disclaimer

A. Search terms and notes

I expanded your previous search by adding additional terms for mechanical alignment, broadening some of the terms and searching in the keyword heading field in addition to the title/abstract fields.

I ran the search in 3 databases - Medline (Ovid), Embase (Ovid) and CENTRAL (Cochrane). I did not run the search in Pubmed as our Medline subscription includes Pubmed articles, however if you would prefer me to run this as a separate search please let me know.

Date range: All dates

Limits: No limits used

For full search strategy see Section D below.

Please acknowledge this work in any resulting paper or presentation as:
Evidence search: Kinematic vs mechanical alignment in total knee replacement. Naomi Majek. 23rd October, 2025. UK: Stockport NHS Knowledge and Library Services.

B. How to access full content

Links are given to full text resources where available. For some of the papers, you will need an **NHS OpenAthens Account**. If you do not have an account you can register online.

You can then access the papers by simply entering your username and password. If you do not have easy access to the internet to gain access, please let us know and we can download the papers for you.

C. Search results

D. Search strategy

Ovid MEDLINE(R) ALL <1946 to October 22, 2025>

1 "Kinematic alignment".ti,ab,kf. 516
2 "Kinematically aligned".ti,ab,kf. 212
3 KA.ti,ab,kf. 30342
4 (Kinematic* adj3 alignment).ti,ab,kf. 639
5 (Kinematic* adj3 aligned).ti,ab,kf. 233
6 1 or 2 or 3 or 4 or 5 30794
7 "mechanical alignment".ti,ab,kf. 924
8 "mechanically aligned".ti,ab,kf. 227
9 MA.ti,ab,kf. 100494
10 (mechanical* adj3 alignment).ti,ab,kf. 1369
11 (mechanical* adj3 aligned).ti,ab,kf. 324
12 (neutral adj2 alignment).ti,ab,kf. 648
13 "conventional alignment".ti,ab,kf. 70
14 "traditional alignment".ti,ab,kf. 84
15 7 or 8 or 9 or 10 or 11 or 12 or 13 or 14 102484
16 Arthroplasty, Replacement, Knee/ 36587
17 "total knee replacement".ti,ab,kf. 7604
18 (knee adj2 replacement).ti,ab,kf. 12891
19 "Knee Arthroplasty".ti,ab,kf. 38955
20 "total knee arthroplasty".ti,ab,kf. 31407
21 TKA.ti,ab,kf. 20827
22 TKR.ti,ab,kf. 2928
23 16 or 17 or 18 or 19 or 20 or 21 or 22 54323
24 6 and 15 and 23 361

Embase <1974 to 2025 October 21>

1 "Kinematic alignment".ti,ab,kf. 545
2 "Kinematically aligned".ti,ab,kf. 225
3 KA.ti,ab,kf. 20381
4 (Kinematic* adj3 alignment).ti,ab,kf. 700
5 (Kinematic* adj3 aligned).ti,ab,kf. 254
6 1 or 2 or 3 or 4 or 5 20886
7 "mechanical alignment".ti,ab,kf. 1021
8 "mechanically aligned".ti,ab,kf. 259
9 MA.ti,ab,kf. 122995
10 (mechanical* adj3 alignment).ti,ab,kf. 1541
11 (mechanical* adj3 aligned).ti,ab,kf. 364
12 (neutral adj2 alignment).ti,ab,kf. 787
13 "conventional alignment".ti,ab,kf. 79
14 "traditional alignment".ti,ab,kf. 92
15 7 or 8 or 9 or 10 or 11 or 12 or 13 or 14 125282
16 exp total knee arthroplasty/ 45544
17 exp knee replacement/ 54142
18 exp knee arthroplasty/ 71109
19 "total knee replacement".ti,ab,kf. 11437
20 (knee adj2 replacement).ti,ab,kf. 19716
21 "Knee Arthroplasty".ti,ab,kf. 46941
22 "total knee arthroplasty".ti,ab,kf. 37490
23 TKA.ti,ab,kf. 24502
24 TKR.ti,ab,kf. 4782
25 16 or 17 or 18 or 19 or 20 or 21 or 22 or 23 or 24 77461
26 6 and 15 and 25 384

CENTRAL (Cochrane Library) Date Run: 23/10/2025 14:24:48

ID Search Hits
#1 ("Kinematic alignment"):ti,ab,kw 93
#2 ("Kinematically aligned"):ti,ab,kw 30
#3 (KA):ti,ab,kw 986
#4 (Kinematic* NEAR/3 alignment):ti,ab,kw 120
#5 (Kinematic* NEAR/3 aligned):ti,ab,kw 35
#6 #1 OR #2 OR #3 OR #4 OR #5 1065
#7 ("mechanical alignment"):ti,ab,kw 176
#8 ("mechanically aligned"):ti,ab,kw 47
#9 (MA):ti,ab,kw 9596
#10 (mechanical* NEAR/3 alignment):ti,ab,kw 235
#11 (mechanical* NEAR/3 aligned):ti,ab,kw 51
#12 (neutral NEAR/2 alignment):ti,ab,kw 93
#13 ("conventional alignment"):ti,ab,kw 9
#14 ("traditional alignment"):ti,ab,kw 5
#15 #7 OR #8 OR #9 OR #10 OR #11 OR #12 OR #13 OR #14 9865
#16 MeSH descriptor: [Arthroplasty, Replacement, Knee] explode all trees 4142
#17 ("total knee replacement"):ti,ab,kw 2851
#18 (knee NEAR/2 replacement):ti,ab,kw 7291
#19 ("Knee Arthroplasty"):ti,ab,kw 9105
#20 ("total knee arthroplasty"):ti,ab,kw 7756
#21 (TKA):ti,ab,kw 4541
#22 (TKR):ti,ab,kw 918
#23 #16 OR #17 OR #18 OR #19 OR #20 OR #21 OR #22 12047
#24 #6 AND #15 AND #23 106

E. Disclaimer

We hope that you find the evidence search service useful. Whilst care has been taken in the selection of the materials included in this evidence search, the Library and Knowledge Service is not responsible for the content or the accuracy of the enclosed research information. Accordingly, whilst every endeavour has been undertaken to execute a comprehensive search of the literature, the Library and Knowledge Service is not and will not be held responsible or liable for any omissions to pertinent research information not included as part of the results of the enclosed evidence search. Users are welcome to discuss the evidence search findings with the librarian responsible for executing the search. We welcome suggestions on additional search strategies / use of other information resources for further exploration. You must not use the results of this search for commercial purposes. Any usage or reproduction of the search output should acknowledge the Library and Knowledge Service that produced it. Search outputs may be published on KnowledgeShare for circulation to other healthcare staff and students. The original requester's name is not associated with the output unless permission is given.

Please acknowledge this work in any resulting paper or presentation as:
Evidence search: Kinematic vs mechanical alignment in total knee replacement. Naomi Majek. 23rd October, 2025. UK: Stockport NHS Knowledge and Library Services.
